# Association of Aspirin Use in Primary Prevention and Cardiovascular Events: A Retrospective Analysis of the VITAL Cohort

**DOI:** 10.3390/jpm15030089

**Published:** 2025-02-26

**Authors:** Daniel Caldeira, Mariana Alves, Nilza Gonçalves, João Costa, Joaquim J. Ferreira, Fausto J. Pinto

**Affiliations:** 1 Centro Cardiovascular da Universidade de Lisboa—CCUL@RISE, Centro Académico de Medicina de Lisboa (CAML), Faculdade de Medicina, Universidade de Lisboa, 1649-028 Lisbon, Portugal; faustopinto@medicina.ulisboa.pt; 2 Cardiology Department, Hospital Universitário de Santa Maria—ULS Santa Maria (ULSSM), 1649-035 Lisbon, Portugal; 3 Centro de Estudos de Medicina Baseada na Evidência (CEMBE), Faculdade de Medicina, Universidade de Lisboa, 1649-029 Lisbon, Portugal; 4 Laboratory of Clinical Pharmacology and Therapeutics, Faculdade de Medicina, Universidade de Lisboa, 1649-029 Lisbon, Portugal; marianaalves88@gmail.com (M.A.); nilzakarina@gmail.com (N.G.); jcosta.fml@gmail.com (J.C.); joaquimjferreira@gmail.com (J.J.F.); 5 Serviço de Medicina, ULS Santa Maria (ULSSM), 1649-035 Lisboa, Portugal

**Keywords:** cardiovascular disease, primary prevention, aspirin, atherothrombotic disease, antiplatelet therapy

## Abstract

**Background:** Aspirin is part of the therapeutic antithrombotic armamentarium for the management of patients with established clinically relevant atherosclerosis or thrombotic cardiovascular disease. Personalized medicine identifies those who benefit most or face fewer risks from aspirin. The role of aspirin in primary prevention is still debatable. We aimed to assess the risks and benefits of aspirin in this setting, using the data of the prospective VITAL (VITamins and Lifestyle) study. **Methods:** We conducted a retrospective evaluation of the VITAL cohort. In this analysis, participants were split according to aspirin usage. Aspirin use was evaluated regarding all-cause mortality, CV mortality, major cardiovascular event (MACE), myocardial infarction, coronary heart disease, total stroke, and hemorrhagic stroke. The hazard ratios (HRs) and 95% confidence intervals (CIs) were estimated to explore the association between cardiovascular events and aspirin usage. The estimates were adjusted for demographic and clinical variables. **Results:** The aspirin users (*n* = 11,570) were older, more frequently men, the body mass index was higher, and the proportion of smokers was smaller compared with non-users (*n* = 13,927). After adjusting for demographic and clinical variables, aspirin was not identified as a predictor of cardiovascular death (HR 1.17, 95%CI 0.89 to 1.55), major cardiovascular events (HR 1.04, 95%CI 0.89 to 1.22), coronary heart disease (HR 1.16, 95%CI 0.98 to 1.37), nor stroke (HR 1.01, 95%CI 0.77 to 1.31). **Conclusion:** In this retrospective analysis of the VITAL cohort, aspirin was not associated with a reduced risk of cardiovascular mortality or events.

## 1. Introduction

Antiplatelet treatment is a cornerstone in the management of patients with established atherosclerotic cardiovascular disease (ASCVD), effectively reducing the risk of recurrent cardiovascular events. Aspirin, the most common antiplatelet agent, acts by inhibiting the production of thromboxane, a molecule responsible for platelet aggregation and thrombus formation. Aspirin is widely available without a prescription, typically as 300 mg tablets, and is commonly used as an anti-inflammatory drug for minor aches, pains, and fever reduction. Low-dose aspirin (usually 75 mg) is widely used in cardiovascular and cerebrovascular prevention, helping to reduce the risk of heart attacks, strokes, and blood clots in high-risk individuals. In addition, aspirin is used in obstetric care to prevent complications such as pre-eclampsia in pregnant women at elevated risk [1].

Antiplatelet drugs are recommended for the secondary prevention of cardio- and cerebrovascular disease. However, its role in primary prevention remains controversial and challenging to define [2,3,4]. This controversy arises from the delicate balance between its modest cardiovascular benefits and the potential for serious adverse events, particularly bleeding complications. Contemporary meta-analyses have demonstrated that while aspirin use in primary prevention may yield a small reduction in cardiovascular events, the accompanying increase in bleeding risk often outweighs the benefits, resulting in an uncertain net clinical benefit [5,6,7,8,9,10].

For primary prevention, these drugs are advised only in patients at very high risk but are not mandatory, as the balance between risk and benefit remains unsettled. In older individuals, the age-related risk of bleeding necessitates a careful risk/benefit evaluation before prescribing antiplatelet drugs, whether for primary or secondary cardiovascular prevention [11].

In light of these findings, the guidelines from major cardiovascular societies have taken a cautious stance. The 2019 American College of Cardiology (ACC)/American Heart Association (AHA) guidelines recommend the infrequent use of aspirin in individuals without established ASCVD, given the lack of a clear net benefit [12]. Similarly, the ESC guidelines for cardiovascular disease prevention advise against the routine use of antiplatelet therapy in individuals with low or moderate cardiovascular risk due to concerns over major bleeding, assigning a class III recommendation with level A evidence [2]. These positions underscore the need for a more nuanced, patient-centered approach to aspirin therapy in primary prevention, taking into account the individual risk factors for both cardiovascular events and bleeding.

Personalized medicine approaches, integrating tools such as risk stratification scores and biomarker profiles, may help refine the identification of patients most likely to benefit from antiplatelet therapy [13]. However, real-world data on this issue remain limited.

To address this knowledge gap, we conducted a post hoc analysis of the VITAL (VITamins and Lifestyle) cohort data, aiming to evaluate whether findings from randomized controlled trials regarding aspirin use are applicable to broader, non-trial populations. This analysis seeks to inform the ongoing debate surrounding aspirin therapy in primary prevention by providing insights into its benefits and risks in a real-world cohort.

## 2. Materials and Methods

This study utilized retrospective data from the VITAL (VITamins and Lifestyle) cohort in a post hoc evaluation, made available through the Project Data Sphere platform (https://doi.org/10.34949/n4c7-zm25, accessed on 26 March 2024).

The VITAL study is a large, prospective cohort designed to investigate the associations between supplement use, cancer risk, and cardiovascular outcomes. Originally conducted in western Washington state, the study recruited participants between 2000 and 2002 and targeted men and women aged 50–76 years.

The cohort comprised 77,738 participants who completed detailed baseline questionnaires on supplement use, diet, and other cancer and cardiovascular risk factors. Additionally, 70% of participants provided self-collected buccal cell specimens for DNA analysis, enhancing the study’s potential for genetic and biomarker-based evaluations. However, these data were not utilized in our analysis.

In the original randomized controlled VITAL trial initiated in 2010, 25,871 participants were followed for a median of 5.3 years. The trial used a 2 × 2 factorial design to assess the impact of vitamin D (2000 IU/day) and/or omega-3 fatty acid (1 g/day) supplementation on primary prevention of cancer and cardiovascular events.

### 2.1. Participants

The participants in the VITAL study were carefully selected to exclude individuals with a history of major cardiovascular or cancer events, including myocardial infarction, stroke, transient ischemic attack (TIA), coronary-artery bypass grafting (CABG), percutaneous coronary intervention, and cancer other than non-melanoma skin cancer.

Further exclusion criteria included renal failure requiring dialysis, hypercalcemia, parathyroid dysfunction, liver cirrhosis, and granulomatous diseases. Additionally, participants were asked to limit their use of supplemental vitamin D and calcium and abstain from fish oil supplements. These stringent inclusion and exclusion criteria ensured that the study focused on a healthy, at-risk population free from significant pre-existing comorbidities.

To assess the long-term outcomes, the study employed an efficient follow-up system using linkages to public databases for tracking cancer diagnoses, cardiovascular events, deaths, and changes in residence. This robust methodology minimized loss to follow-up and ensured comprehensive outcome capture [14].

### 2.2. Outcomes

The data collection included baseline and follow-up questionnaires administered at 6 months, 12 months, and annually thereafter (for 6 years). In the randomized controlled component of the VITAL trial, the primary outcomes were cancer incidence and major cardiovascular adverse event (MACE), which included myocardial infarction, stroke, and death from cardiovascular causes. Cardiovascular deaths were confirmed using hospital records, autopsy reports, and death certificates, with outcome adjudication performed by a blinded committee of physicians [14].

In this retrospective analysis, we explored the associations between aspirin use and several cardiovascular outcomes, including MACE—as defined before—expanded MACE (including MACE and coronary revascularization such as percutaneous coronary intervention or coronary artery bypass grafting), total myocardial infarction (MI), total coronary heart disease (CHD) (which included non-fatal MI, coronary revascularization, and CHD-related deaths), and total stroke and hemorrhagic stroke, classified according to the Trial of Org 10,172 in Acute Stroke Treatment (TOAST) criteria. To enhance reliability, outcomes such as myocardial infarction and stroke were defined using established clinical guidelines, including the Joint European Society of Cardiology/American College of Cardiology Foundation/American Heart Association/World Heart Federation criteria for myocardial infarction and the TOAST criteria for stroke [15,16,17].

### 2.3. Supplement Use and Confounding Factors

The VITAL study targeted a population with a high prevalence of supplement use. At baseline, 66% of participants reported regular multivitamin use, while individual vitamin C, vitamin E, and calcium use was reported by approximately 46–47% of the cohort. The participants reported an average duration of supplement use spanning 5–8 years over the previous decade [15].

Confounding factors were rigorously assessed, with strong associations observed between supplement use and other health behaviors, including regular nonsteroidal anti-inflammatory drug use, higher intake of fruits and vegetables, and increased recreational physical activity. These associations, although robust, presented challenges in disentangling direct effects from lifestyle influences [14,15].

### 2.4. Statistical Methods

Participants were stratified based on baseline aspirin usage into aspirin users and non-users. The descriptive statistics were calculated, with means and standard deviations used for continuous variables and absolute and relative frequencies for categorical variables.

Comparisons between groups were performed using independent sample t-tests for continuous variables and chi-square tests for categorical variables. To evaluate the association between aspirin use and the outcomes of interest, univariate and multivariate Cox proportional hazards regression models were employed. The hazard ratios (HRs) and their corresponding 95% confidence intervals (95%CIs) were reported for each outcome.

The tied events were managed using the Breslow method. The assumption of proportional hazards was tested using Schoenfeld residuals to ensure the validity of the Cox regression models. The statistical analyses were conducted using Stata/SE software version 17.0 (StataCorp), with a *p*-value < 0.05 considered indicative of statistical significance.

This robust statistical approach allowed for the adjustment of potential confounders and ensured that the findings were both reliable and clinically meaningful.

## 3. Results

**Figure 1:**
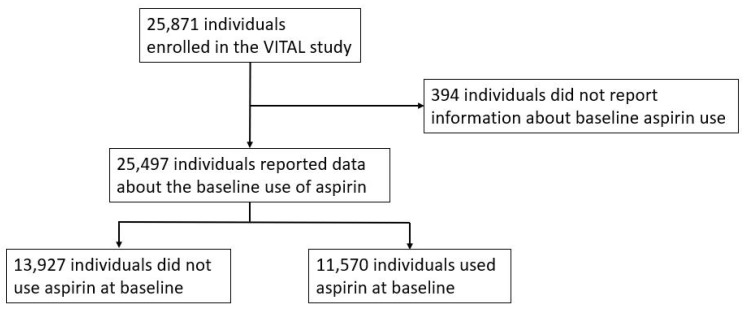
Flowchart of patients from the VITAL (VITamins and Lifestyle) cohort study in analysis.

Out of the 25,871 individuals enrolled in the VITAL study, 25,497 participants provided data on baseline aspirin use, while 394 individuals did not report such information. Among those who reported baseline aspirin use, 13,927 individuals did not use aspirin at baseline, whereas 11,570 individuals reported using aspirin at baseline (Figure 1).

The characteristics of the group of patients taking aspirin included age, sex, body mass index (BMI), race, smoking habits, and comorbidities (Table 1). The aspirin users’ mean age was 67.5 years (vs. 65.9 years in non-users, *p* < 0.001), 54.7% were male (vs. 45.1%, *p* < 0.001), and the mean BMI value was higher in aspirin users with 28.4 kg/m^2^ compared with a mean BMI of 27.8 kg/m^2^ in non-users (*p* < 0.001). The number of current smokers amongst aspirin users was lower compared with aspirin non-users (6.2% versus 8.0%, *p* < 0.001). At baseline, 58.5% of aspirin users had arterial hypertension treated with medication, significantly higher compared with aspirin non-users (42.1%, *p* < 0.001). The aspirin users with diabetes mellitus were more frequent (17.7%) than diabetic individuals not treated with aspirin (10.3%; *p* < 0.001). The usage of cholesterol-lowering medication and diabetes mellitus (DM) medication was also higher for patients under aspirin medication compared with patients who were not using aspirin (Table 1).

Table 2 shows the events and incidence rates within 5.3 years of follow-up according to the use of aspirin at baseline.

**Figure 2:**
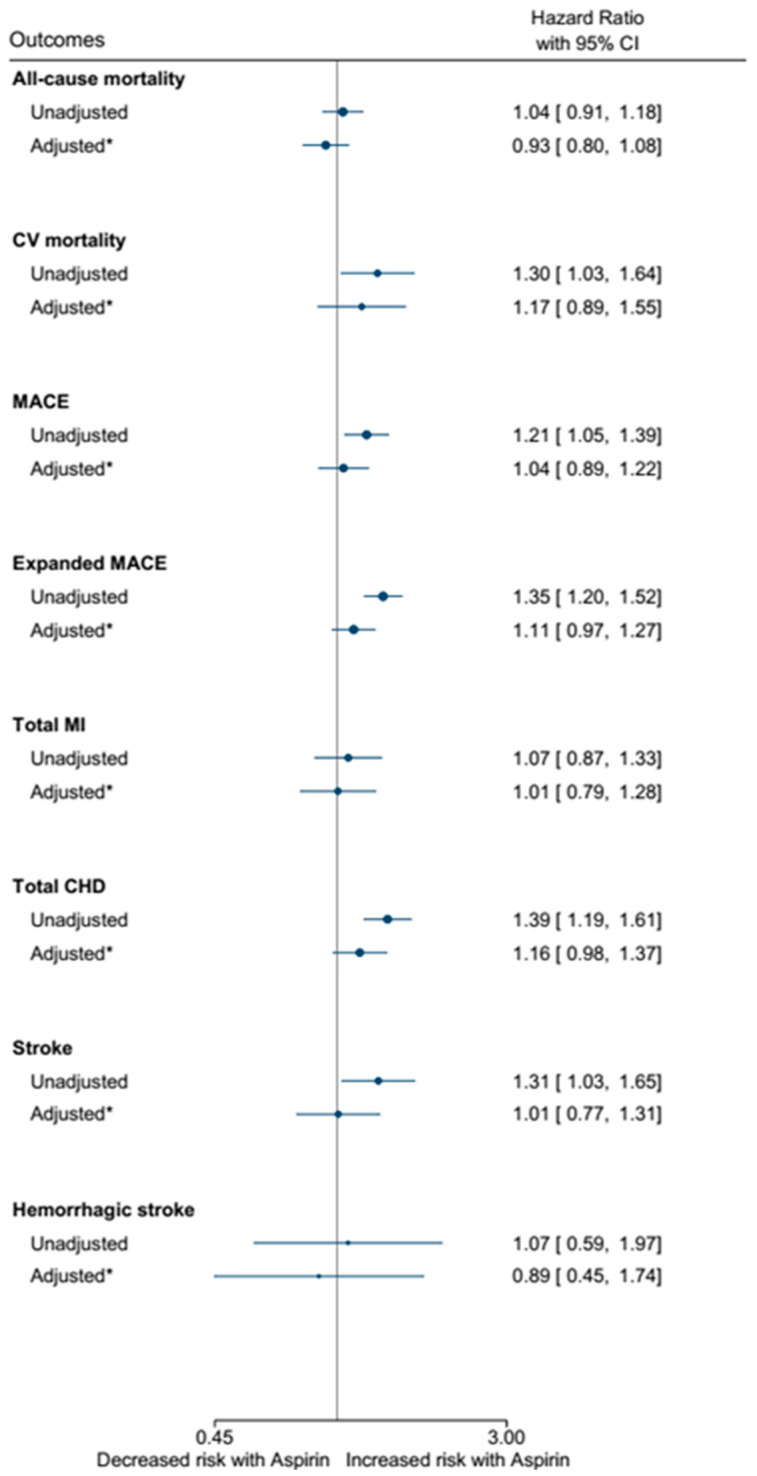
Hazard ratio with 95%CI of death and cardiovascular events unadjusted and adjusted for demographic and clinical variables. * Adjusted for age, sex, race, BMI, smoking, hypertension, parental history of myocardial infarction, diabetes mellitus, and the use of statins.

In the unadjusted analysis, the aspirin group presented a higher risk of cardiovascular mortality (HR 1.30, 95%CI 1.03 to 1.64) and cardiovascular events, such as major cardiovascular events (HR 1.21, 95%CI 1.03 to 1.64), coronary heart disease (HR 1.39, 95%CI 1.19 to 1.65), and stroke (HR 1.31, 95%CI 1.03 to 1.65) (Figure 2). However, after adjustingfor demographic and clinical variables, aspirin was not identified as a predictor of cardiovascular death (HR 1.17, 95%CI 0.89 to 1.55), major cardiovascular events (HR 1.04, 95%CI 0.89 to 1.22), coronary heart disease (HR 1.16, 95%CI 0.98 to 1.37), nor stroke (HR 1.01, 95%CI 0.77 to 1.31)—Figure 2 and Appendix A.

The adjusted HR for aspirin use in primary prevention varied by race/ethnicity (Table 3). Among Caucasians, no significant differences were found for mortality (HR 0.86, 95%CI 0.74–1.04), CV mortality (HR 0.96, 95%CI 0.68–1.34), or MACE (HR 0.96, 95%CI 0.80–1.16). In Black participants, aspirin use was associated with a higher CV mortality (HR 2.01, 95%CI 1.10–3.68), while other outcomes were not significant. Results for Hispanic, Asian, and Native American groups were inconclusive due to wide confidence intervals or insufficient data. These findings suggest potential racial differences in aspirin’s effects, particularly in Black participants, warranting further investigation.

## 4. Discussion

The use of aspirin in primary prevention was not associated with the risk reduction of cardiovascular events, after adjustment for demographic and clinical characteristics, in this post hoc evaluation of the VITAL cohort data. It should be stressed that individuals with prescribed aspirin at baseline had an overall prevalence of clinical characteristics that predict a higher risk of cardiovascular events, such as increased age, male gender, higher BMI, and more frequent parental history of MI. These findings suggest that prescribing practices may have been influenced by clinicians’ perception of risk, aligning with the principles of individualized care.

It is also important to contextualize these findings within the timeframe of data collection, which occurred over a decade ago when the evidence against aspirin in primary prevention was less definitive. This underscores the need for contemporary analyses that incorporate the advancements in personalized medicine, such as the integration of genetic and biomarker-based risk stratification, to guide more precise therapeutic decisions in primary prevention.

In 2009, a meta-analysis of randomized controlled trials suggested that aspirin use could reduce by 12% the risk of vascular events, mainly non-fatal MI, and suggested that aspirin could even have benefits in reducing all-cause mortality [18]. However, it is plausible that these cardiovascular benefits reflected an era when cardiovascular risk factors were less effectively managed, leading to a higher relative benefit of antiplatelet therapy.

Over the past two decades, accumulating evidence has challenged the role of aspirin in primary prevention. Despite this growing evidence, a 2017 National Health Interview Survey revealed that 23.4% of individuals reported using aspirin for primary prevention, with 23% of these doing so without a physician’s recommendation [19]. So, these findings highlight not only the need for continued physician education but also the importance of managing patient expectations and beliefs about the benefits and risks of aspirin. Incorporating personalized medicine approaches, such as individualized risk stratification and shared decision-making, could help bridge the gap between clinical evidence and patient behavior, optimizing the use of aspirin in primary prevention.

The main evidence against aspirin use in primary prevention came to light in 2018 with three important trials—the ASPREE trial (older adults) [20], ASCEND trial (patients with diabetes) [21], and ARRIVE study (average-risk adults) [22]. Subsequently, systematic reviews have reached the same conclusions as our post hoc analysis in the general population, but also in specific groups of higher cardiovascular risk, such as those with diabetes or chronic kidney disease [6,7,8,23]. On the other hand, years after this change in paradigm, few studies still point in a different direction. For instance, Zhao et al. stated that low-dose aspirin should be considered for primary prevention in aboriginal people with high cardiovascular risk, due to reducing all-cause mortality (HR 0.45, 95%CI 0.34 to 0.60) and not significantly increasing bleeding risk (HR 1.13, 95%CI 0.39 to 3.26). We cannot extrapolate this data from people living in remote Australia with a mean age of only 42 years old to other urbanistic and older populations [24]. This underscores the importance of personalized medicine in interpreting and applying clinical evidence.

Building on the principles of personalized medicine, it is essential to explore how factors such as race and ethnicity influence the effectiveness and safety of aspirin, shedding light on potential disparities in its cardiovascular benefits. Contrary to our results, other studies suggest that low-dose aspirin may not offer the same protective benefits for African Americans as it does for other populations. For instance, one study found that low-dose aspirin did not reduce the risk of fatal heart attacks among African Americans but showed a trend toward a decreased risk in white participants, especially women. Additionally, African Americans taking low-dose aspirin experienced an 18% increased risk of dying from a heart event, while non-Hispanic white participants had a 14% decreased risk [25].

These disparities may stem from genetic variations affecting aspirin metabolism, poor control of cardiovascular risk factors, and differences in adherence to aspirin regimens. The medication interactions more common in African American populations could also play a role. Moreover, African Americans have been historically underrepresented in aspirin studies, limiting the applicability of findings to this population [25].

Beyond biological factors, social and cultural influences may impact aspirin use and its outcomes. Positive peer support and cultural beliefs about aspirin have been linked to higher adoption rates in African American communities. The research in other contexts, such as cancer prevention, has also suggested that aspirin’s effects may vary across racial groups, further highlighting the need to explore these differences in cardiovascular prevention [26].

These findings underscore the importance of more inclusive research to better understand the relationship between aspirin use, race/ethnicity, and cardiovascular outcomes. Addressing these disparities can guide the development of tailored prevention strategies and improve outcomes for underrepresented populations.

Another interesting topic regarding cardiovascular prevention is the great interest in simple approaches with wide applicability to address cardiovascular risk factors, including using fixed-dose combination drug regimens, also known as polypills. An individual participant data meta-analysis of three large randomized controlled trials (TIPS-3, HOPE-3, and Polylran) evaluated a fixed-dose combination strategy of at least two blood-pressure-lowering agents plus a statin with or without aspirin. The mean age of the participants was 63 ± 7.1 years old, and the estimated 10-year cardiovascular disease risk was 17.7 ± 8.7%. The group receiving fixed-dose combination strategies including aspirin had a 47% reduction (HR 0·53, 95%CI 0·41 to 0·67; number needed to treat [NNT] 37) in the primary outcome (a composite of cardiovascular death, myocardial infarction, stroke, or arterial revascularization), compared with a 32% reduction (HR 0·68, 95%CI 0·57 to 0·81; NNT 66) for fixed-dose combination strategies without aspirin. Regarding side effects related to aspirin, they were non-statistically different. This bundle strategy seems to be more effective in older patients (>63 years old) with high or very-high CV risk (10-year CV risk > 9.6%) [27].

Despite this interesting putative beneficial effect of polypill regimen in a subgroup of patients, the current evidence of using aspirin alone in primary prevention in the general population does not seem to overcome the bleeding risk.

While our analysis provides valuable insights into this important topic, it is not without limitations. The VITAL trial/cohort, by including individuals who were willing to participate in a clinical trial aimed at reducing their cardiovascular disease (CVD) or cancer risk [28], is subject to selection bias. However, it is noteworthy that almost half of the participants had hypertension, a third were on statin therapy, and 14% were diagnosed with diabetes. These cardiovascular risk factors were well represented in the study population, and the results were independent of these factors.

Another acknowledged limitation of our analysis is its retrospective and post hoc nature. Additionally, the lack of data regarding bleeding risk presents a significant drawback, as only hemorrhagic stroke outcomes were collected, showing no differences between groups. Nevertheless, given that no net benefit was observed with antiplatelet medication in this population, the absence of detailed bleeding risk data becomes less critical in this context.

These limitations highlight the importance of conducting prospective studies that incorporate personalized approaches, such as biomarker- or genetic-based risk stratification, to better define the role of aspirin in primary prevention. Such studies could refine therapeutic strategies and identify subgroups of patients who may derive the greatest benefit while minimizing potential harm.

## 5. Conclusions

This retrospective/post hoc multivariate-adjusted (demographic and clinical characteristics) analysis of participants enrolled in the VITAL cohort did not show an association between aspirin use and reduced cardiovascular events in the setting of primary prevention.

## Figures and Tables

**Table 1:** Patients’ demographic and clinical characteristics according to baseline use of aspirin.

	Aspirin—Yes (11,570)	Aspirin—No (13,927)	*p*-Value
Age (years) (mean, SD)	67.5 (6.9)	65.9 (7.1)	<0.001
Sex (male) (%, n)	54.7 (6330)	45.1 (6282)	<0.001
BMI (kg/m^2^) (mean, SD)	28.4 (5.7)	27.8 (5.7)	<0.001
Race/ethnic group (%, n)			<0.001
Non-Hispanic white	74.8 (8446)	68.6 (9370)	
Black	17.3 (1950)	22.4 (3061)	
Hispanic	3.5 (393)	4.4 (603)	
Asian	1.3 (147)	1.7 (235)	
Native American or Alaskan	1.0 (118)	0.8 (107)	
Other/unknown	2.1 (241)	2.0 (273)	
Current smoking (%, n)	6.2 (711)	8.0 (1113)	<0.001
Parental history of MI (%, n)	17.8 (1837)	14.3 (1765)	<0.001
Hypertension treated with medication (%, n)	58.5 (6277)	42.1 (5827)	<0.001
Diabetes mellitus at baseline (%, n)	17.7 (2047)	10.3 (1433)	<0.001
Baseline use of statins (%, n)	45.8 (5258)	25.8 (3571)	<0.001

SD—standard deviation; BMI—body mass index; MI—myocardial infarction.

**Table 2:** Number of events and incidence of outcomes according to aspirin use at baseline (follow-up period 5.3 years).

Outcomes	Events n (%)		Incidence per 1000-PY (95%CI)	
	Aspirin	No Aspirin	Aspirin	No Aspirin
All-cause mortality	443 (3.8%)	508 (3.6%)	7.2 (6.6–7.9)	6.9 (6.4–7.6)
Cardiovascular mortality	146 (1.3%)	134 (1.0%)	2.4 (2.0–2.8)	1.8 (1.5–2.2)
MACE	396 (3.4%)	391 (2.8%)	6.5 (5.9–7.2)	5.4 (4.9–6.0)
Expanded MACE	568 (4.9%)	506 (3.6%)	9.5 (8.7–10.3)	7.0 (6.4–7.6)
Total MI	160 (1.4%)	178 (1.3%)	2.6 (2.3–3.1)	2.4 (2.1–2.8)
Total CHD	358 (3.1%)	310 (2.2%)	5.9 (5.3–6.6)	4.3 (3.8–4.8)
Total stroke	148 (1.3%)	135 (1.0%)	2.4 (2.1–2.9)	1.9 (1.6–2.2)
Hemorrhagic stroke	20 (0.2%)	22 (0.2%)	0.33 (0.21–0.51)	0.30 (0.20–0.46)

**Table 3:** Adjusted hazard ratios (HRs) for all-cause mortality, cardiovascular mortality, and major adverse cardiovascular event (MACE), comparing aspirin vs. no aspirin, stratified by race/ethnicity.

	Mortality	CV Mortality	MACE
Caucasian	0.86 (0.74–1.04)	0.96 (0.68–1.34)	0.96 (0.80–1.16)
Black	1.13 (0.80–1.61)	2.01 (1.10–3.68)	1.31 (0.86–1.98)
Hispanic	0.78 (0.35–1.73)	0.22 (0.02–2.01)	1.00 (0.40–2.47)
Asian	1.80 (0.11–29.90)	N/A *	1.16 (0.22–6.27)
Native American	1.30 (0.29–5.84)	2.14 (0.52–8.76)	0.33 (0.05–2.25)

* No events.

**Figure A1:**
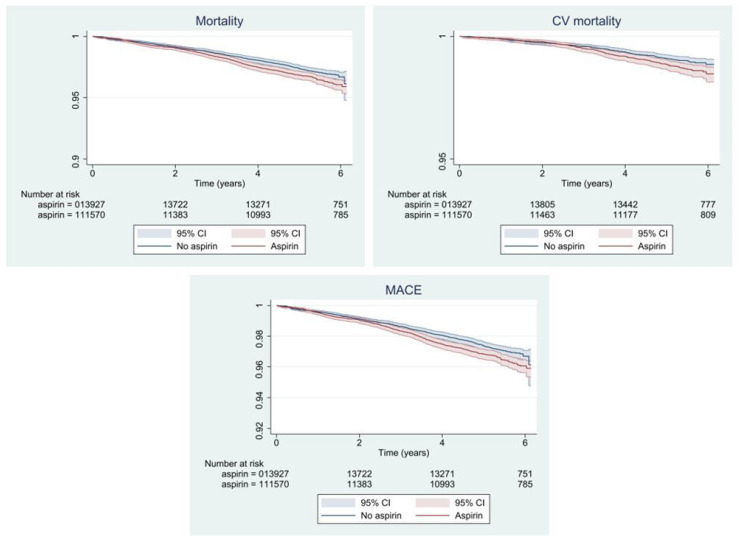
Kaplan-Meier curves for all-cause mortality, cardiovascular mortality, and major adverse cardiovascular event (MACE).

## Data Availability

The original data presented in the study are available in the Project Data Sphere platform (https://doi.org/10.34949/n4c7-zm25).

## Abbreviations

The following abbreviations are used in this manuscript:

ASCVD	atherosclerotic cardiovascular disease
ACC	American College of Cardiology
AHA	American Heart Association
ESC	European Society of Cardiology
VITAL	VITamins and Lifestyle
TIA	transient ischemic attack
CABG	coronary-artery bypass grafting
MACE	major cardiovascular adverse event
MI	myocardial infarction
CHD	coronary heart disease
TOAST	Trial of Org 10172 in Acute Stroke Treatment
HR	hazard ratio
CI	confidence interval
CVD	cardiovascular disease
NNT	number needed to treat
